# Ion and Molecule Transport in Membrane Systems

**DOI:** 10.3390/ijms22073556

**Published:** 2021-03-30

**Authors:** Victor Nikonenko, Natalia Pismenskaya

**Affiliations:** Membrane Institute, Kuban State University, Krasnodar 350040, Russia; n_pismen@mail.ru

Membranes plays enormous role in our life. A biological cell membrane is an enclosing film that acts as a selective barrier within or around the cell and controls the fluxes of substances in and out of cells. Artificial membranes are widely used for the treatment of water and food solutions like milk, juice and wine); fractionation of organic acids, bioactive compounds and nutrients; and energy production among other applications. Artificial membranes largely mimic the structure and functions of biological membranes. Like cell membranes, many kinds of artificial membranes involve macromolecules consisting of a relatively long hydrophobic polymer chain and a hydrophilic “head” at its end. Such elements allow multiple types of interactions (hydrophobic–hydrophobic, dipole–dipole, ion–dipole, ion–ion) between them and water, which provides a self-assembly resulting in the formation of permselective thin films. Similarity in structure leads to similarity in properties and the approaches to studying the laws governing the behavior of both biological and artificial membranes. It is of interest that Kedem and Katchalsky deduced their famous equations for the description of transport processes in biological membranes [1,2]. Now these equations are largely used for all types of membranes, in particular for modelling ion and water transport in a promising technological process named Pressure Retarded Osmosis (PRO), which is employed for energy harvesting from salinity variations [3].

The idea of this special issue is to recollect the papers describing physico-chemical and chemico-physical aspects of ion and molecule transport, which are common for both biological and artificial membrane systems. The scope of the issue involves experimental studies and mathematical modeling to provide new knowledge on the mechanisms of ion and molecule transport in artificial and living systems; similarities in behavior of biological and artificial membranes; biomimetic structural features of artificial membranes and their impact on membrane properties and performance for separation processes; generalities and case studies in the field of material structure–properties relationships; thermodynamics and irreversible thermodynamics description; equilibriums and kinetics of transport processes in membrane systems; the coupling of ion and molecule transport with chemical reactions and catalysis; impact of forced and natural convection on ion and molecule transport; mechanisms of electric current-induced convection and its impact on ion and molecule transport across membranes; concentration polarization and coupled effects occurring in membrane systems under the action of external pressure and electric driving forces; physico-chemical and chemico-physical aspects of all kinds of separation, purification, and fractionation in membrane systems. In all cases, the analysis of phenomena at molecular level is encouraged.

Within this issue, there are papers devoted to the study of thin mechanisms of ionic and molecular transport in cell membranes [4,5,6], as well as artificial ones, that mimic biological membranes [7,8]. Y. Trofimov et al. reported the results of molecular dynamic simulations of water confined in the pore of a cell membrane, taking into account that the microscopic properties of water near the molecular surface are radically different from those in the bulk solution [4]. K. Yue et al. [6] applied molecular dynamics to simulate the interactions of inhaled pollutant nanoparticles with a pulmonary surfactant monolayer. The review by M. Tingey et al. [5] evaluated the current tools and methodologies available to study the role of transmembrane proteins in some kinds of cell membranes. The team of Y. Zhang et al. [7] described the mechanism of selective separation of volatile fatty acids (VFAs) using polymer inclusion membranes (PIMs) containing ionic liquids as the carrier. This process mimics the selective transport of compounds like phenols and amino acids by facilitating diffusion through cell membranes. W. Tien et al. [8] described the effects of cholesterol on the water permittivity of biomimetic ion-pair amphiphile bilayers, which are used to fabricate vesicles for various pharmaceutical applications.

The problems of preparation and the properties of artificial membranes are considered in [9,10,11,12,13]. S. Aziz et al. [9] developed polymer-blend electrolyte membranes based on chitosan, a biopolymer. It is shown that new membranes have a high performance in electrical double-layer capacitor applications, such as water desalination. The use of the Truhan model permitted a detailed analysis of ion transport parameters of the chitosan-based polymer membrane [10]. B. Jaleh et al. [11] used deposition of TiO_2_ nanoparticles to improve wettability of an O_2_ plasma-activated polypropylene membrane. A new hydroxide exchange membrane was synthesized by A. Abbasi et al. [12]. Low zincate crossover and high discharge capacity of this kind of membrane makes it promising for use in zinc–air batteries, an alternative to lithium–ion batteries for various applications. Novel anion-exchange membranes combining the advantages of a dense functionalization architecture and crosslinking structure were fabricated by Q. Ge et al. [13]. A high ratio of hydroxide conductivity to water swelling suggests that these membranes have high potential for application in fuel cells.

The analysis of some membrane properties that affect overall performances was made in references [14,15]. F. Luo et al. [14] examined the transmembrane potential across ion exchange membranes to evaluate their possible power efficiency when applied in a “reverse electrodialysis heat engine”. The impact of different ion composition of a salt solution containing NaHCO_3_, Na_2_CO_3_, and NH_4_Cl electrolytes was examined. The performance of another membrane process, important for the recovery of fertilizers from wastewater, was studied by O. Rybalkina et al. [15]. Phosphorus transport through anion-exchange membranes in the course of electrodialysis of an NaH_2_PO_4_ solution was investigated. It was shown that when H_2_PO_4_^−^ ions enter the membrane, a part of these anions dissociates, resulting in the parasitic transport of H^+^ ions in the depleted solution, which essentially reduces current efficiency of the process.

The analysis of interesting and diverse applications of artificial membranes is reported in references [16,17,18,19]. An ionic liquid-driven supported liquid membrane system was applied by J. Li et al. [16] for preparing a special kind of phosphors, which were characterized by good luminescent properties. The team of L. Bazinet for the first time realized simultaneous separation of peptides from salmon protein hydrolysate by three ultrafiltration membranes stacked in an electrodialysis system [17]. A thorough study of this green and ultraselective process is presented. An application concerning the processing of whey was investigated in references. [18,19]. Dufton et al. [18] used a special mode of electrodialysis where Pulsed Electric Fields (PEFs) were applied. It was found that the PEF mode, in which current pulses alternate with zero-current pauses, can increase the degree of both demineralization and deacidification of the whey, as well as reduce membrane scaling. Another kind of electric current pulse, Pulsed Electrodialysis Reversal (PER), was applied by A. Merkel and A. Ashrafi [19] for the demineralization of acid whey. They alternated relatively long pulses of direct current with short pulses of reverse polarity to decrease the fouling onto the membrane surface during electrodialysis (ED). It was found that the fouling on the diluate side of both the cation and anion exchange membranes in a PER regime was reduced compared to the conventional ED. 

The issues connected with the hydrodynamic conditions of mass transfer in membrane systems are considered in references. [20,21,22]. G. Battaglia et al. [20] studied the effect of transmembrane pressure (TMP), which may arise in membrane stacks for electrodialysis (water desalination) or reverse electrodialysis (energy production by salinity gradient), or on solution flow and mass transfer. A clear and intelligible review of the effect of profiling ion-exchange membranes on the properties of electrodialysis was presented by S. Pawlowski, J. Crespo and S. Velizarov [21]. They were very convincing when showing that there is exciting potential for improving membrane performance because of the enormous degree of freedom for creating new profile geometries on a membrane surface. Some problems of the electrokinetic instability of a solution adjacent to an ion-exchange membrane are considered by P. Magnico [22]. The electrokinetic behavior of cation-exchange resin particles arranged in a well-defined geometrical structure was studied by the team led by Z. Slouka [23]. The understanding of the effect of coupling between water and ion transport is of the utmost importance for improving the performance of electro-driven membrane separation processes.
